# Response of legal and illegal cigarette prices to a tax increase in Ethiopia

**DOI:** 10.1136/tc-2023-057931

**Published:** 2023-04-26

**Authors:** Sisay Derso Mengesha, Hana Ross

**Affiliations:** 1 Environmental Health and Noninfectious Disease Research Unit, Ethiopian Public Health Institute, Addis Ababa, Ethiopia; 2 School of Economics, University of Cape Town, Rondebosch, South Africa

**Keywords:** Price, Economics, Illegal tobacco products, Taxation

## Abstract

**Background:**

In 2020, Ethiopia passed a landmark tax proclamation implementing an evidence-based mixed excise system aimed at curbing tobacco use. This study evaluates the impact of the tax increase of more than 600% on both legal and illegal cigarette prices in order to gauge the impact of the tax reform in the presence of a sizeable illicit cigarette market.

**Methods:**

Data on 1774 cigarette prices were obtained from retailers during Empty Cigarette Pack Surveys in the capital and major regional cities conducted in 2018 and 2022. Packs were categorised as ‘legal’ or ‘illicit’ using criteria from the tobacco control directives. Descriptive and regression analyses were used to study the cigarette price changes during the period of 2018–2022, capturing the impact of the 2020 tax increase.

**Result:**

Prices of both legal and illegal cigarettes increased in response to the tax increase. In 2018, the stick prices ranged from ETB0.88 (Ethiopian birr) to ETB5.00 for legal cigarettes while they ranged from ETB0.75 to ETB3.25 for illegal ones. In 2022, a legal stick sold for ETB01.50–ETB2.73 and an illegal stick for ETB1.92–ETB8.00. The average real price of legal and illegal brands increased by 18% and 37%, respectively. The multivariate analysis confirms that prices of illicit cigarettes grew faster compared with the legal ones. By 2022, illicit brands were on average more expensive compared with their legal counterparts. This result is statistically significant at p<0.01.

**Conclusion:**

The prices of both legal and illegal cigarettes increased following the 2020 tax increase, with the average real cigarette price increasing by 24%. As a result, the tax increase likely had a positive impact on public health despite a sizeable illicit cigarette market.

**WHAT IS ALREADY KNOWN ON THIS TOPIC**

- Tobacco tax increases are the most effective measure to reduce tobacco use.
- In 2020, Ethiopia changed its excise tax structure and substantially increased the cigarette tax rate.
- The cigarette market in Ethiopia had a significant illicit cigarette market at the time of the tax increase.

**WHAT THIS STUDY ADDS**

- This is the first study to evaluate the response of legal and illicit cigarettes to the implementation of mixed excise tax regime and an increase in the tax rate in Ethiopia.
- Even though prices of both legal and illegal cigarettes increased, the prices of illegal cigarettes increased more.
- In just 4 years, the market has many new illicit brands.
- Since there is no inflation adjustment in the specific component of the tax, its value has deteriorated greatly over time, and cigarettes in Ethiopia are still affordable.

**HOW THIS STUDY MIGHT AFFECT RESEARCH, PRACTICE OR POLICY**

- The impact of the tax reform on cigarette prices in Ethiopia has not been evaluated. This comprehensive law, which requires mixed tax systems of 30% ad valorem and 8 birr as a specific tax per pack of cigarette, was implemented in February 2020.

## Introduction

 The consumption of tobacco in any form, including legal and illegal cigarette products, imposes social, health and economic harm.^1 2^ Despite a relatively low smoking prevalence in Ethiopia,^3^ tobacco use results in 16 800 premature deaths annually, while causing many tobacco-related illnesses like cancer, heart disease and cardiovascular and chronic respiratory conditions.^4^ Non-communicable diseases cost Ethiopia about 1.84% of its gross domestic product (GDP), equivalent to ETB31 billion (Ethiopian birr) per year,^5^ while the annual economic costs of smoking are around ETB1.4 billion or US$43.6 million.^4^ In addition to the macrolevel damage, buying tobacco denies families the resources they need to escape poverty.^6 7^ In 2020, close to 12% of per-capita GDP was required to purchase 2000 cigarette sticks of the most sold cigarette brand.^8^

Tobacco tax increases, which significantly increase prices of tobacco products, are the most effective measure to reduce tobacco use.^9 10^ Large tax and price increases prevent tobacco initiation, promote cessation and reduce overall tobacco consumption.^11^,^13^ Higher taxes and prices are particularly effective in reducing tobacco use in vulnerable populations, including youth and lower income people, given that these groups are more price sensitive.^1 14^

On 13 February 2020, the Ethiopian Parliament approved the Excise Tax Proclamation No 1186/2020. It introduced a mixed tax structure and higher tax rates. The cigarette excise tax consists of an *ad valorem* component (30% of market price based on ex-factory price plus profit) and a specific component of ETB8 (equivalent to US$0.25 in 2020) on each cigarette pack.^15^ This change led to a 686% increase in cigarette excise tax.^8^

Before the Proclamation was approved, cigarettes in Ethiopia were among the cheapest in the world, even compared with other African countries.^16^ Global Adult Tobacco Survey Ethiopia estimated that the median price of a pack of 20 cigarettes was ETB18.3 in 2016 or about US$0.82.^3^

WHO estimated that the new legislation would increase the tax share of the average retail price of cigarettes from 33% to approximately 54%.^17^ The tax increase was expected to reduce the rate of cigarette smoking among adults by as much as 10% and reduce the number of deaths attributable to smoking by approximately 91 000 people. In addition, tax revenue was expected to rise by as much as 81%, amounting to an additional ETB925 million (US$28.7 million).^17^ Another study predicted an almost 8 million years of life saved in the current population, an increase in tax revenues by US$26 million in the first year after the tax increase and up to 173 000 catastrophic health expenditure cases averted due to lower out-of-pocket expenditures on medical treatment.^18^ These predictions were vulnerable to the industry response to the tax increase.

Established in 1942, the National Tobacco Enterprise (NTE) was a government-owned business until privatisation in 2017.^19 20^ The NTE is now owned by Japan Tobacco International with a 71% share, and by the Yemen-based Sheba Company with a 29% share.^20^ The NTE is the only entity in the country that manufactures, imports and distributes tobacco products. This gives it a monopolistic power to set prices, thus controlling the impact of the tax reform on public health. Following its privatisation, the NTE continues to enjoy a close relationship with the Ethiopian government, allowing it to be directly involved in policy making.^21^ The industry opposes tobacco taxes by publicising inflated and unreliable estimates of the size of the illicit cigarette market, blaming tax policy for that outcome.^22 23^ As a result, there is reluctance on the part of the government to implement annual tax increases as envisioned in the 2020 Excise Tax Proclamation.

This study examines the responses of cigarette prices to the 2020 tax increase. Given the sizeable illicit cigarette market in Ethiopia,^24^ we study the impact on both legal and illegal cigarettes. This information supports the evidence-based tobacco tax policy making with the goal of improving public health in Ethiopia.

## Methods

**Figure 1:**
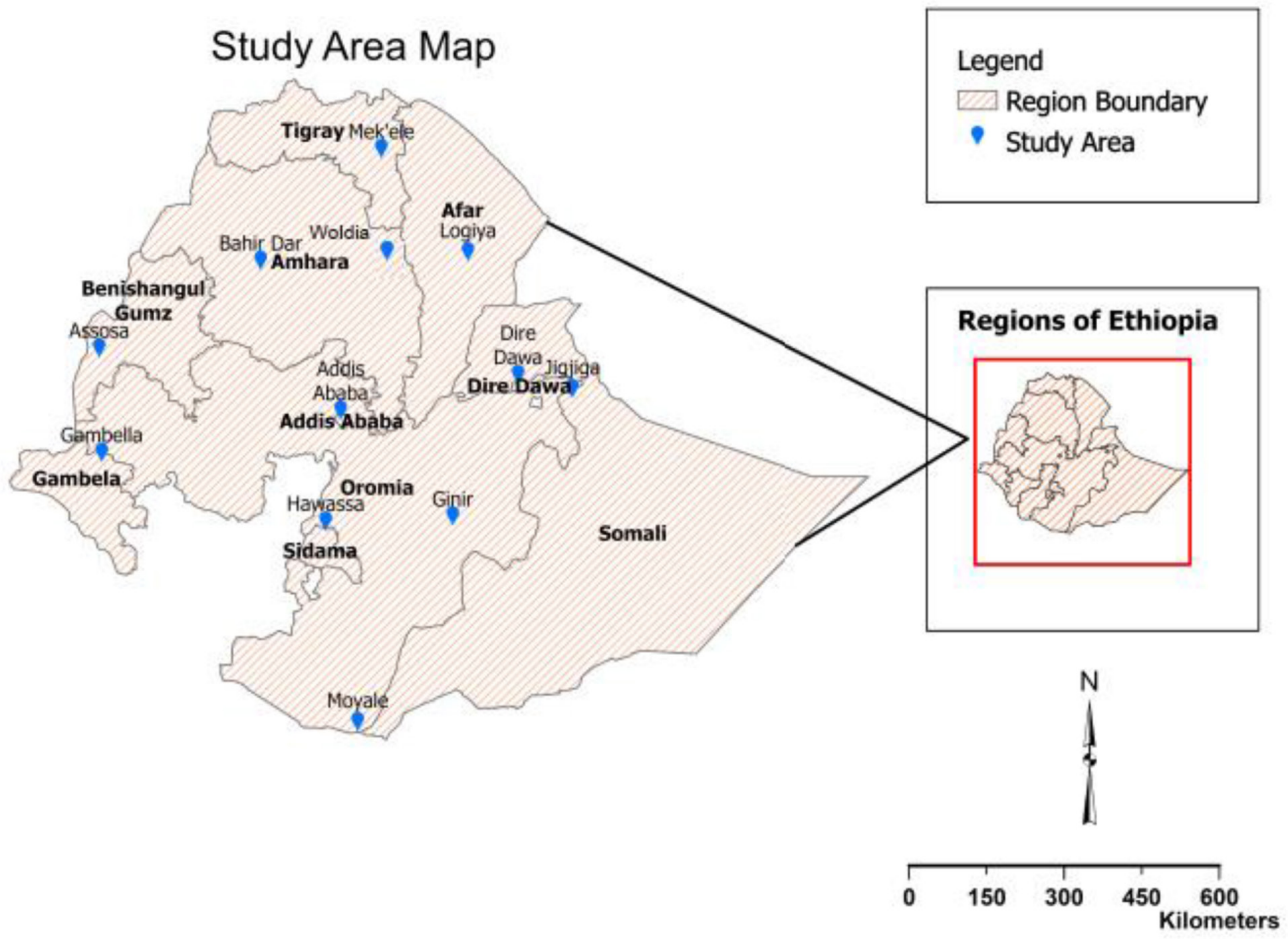
Location of 12 major towns included in the Empty Cigarette Pack Survey (EPS) study.

We used cigarette price data from two Empty Cigarette Pack Surveys (EPS) conducted in 2018 and 2022, respectively. The EPS collected 1774 cigarette prices from retailers located in the capital Addis Ababa and major regional cities: Assosa, Bahir Dar, Dire Dawa, Gambella, Hawassa, Jigjiga, Logiya, Mekele and Moyale. In 2022, the EPS excluded Mekele and Moyale for security reasons and replaced them with Ginir and Woldia (figure 1). Both surveys were observational assessments of cigarette packs from retailers (kiosk and street vendors) and street sources.^25^ All retailers (both those in stores and street vendors) located in randomly selected streets were approached to surrender their empty cigarette packs from 1 day of single cigarette sales. In addition to providing the empty packs, the retailers were interviewed about prices of single sticks as well as packs across cigarette brands they sold. The detailed methodology of the EPS is described elsewhere.^24^

Since there is no tax stamp in Ethiopia, it is not possible to determine if the tax was paid by examining a pack. Therefore, we conservatively classified all domestic cigarette brands (Nyala, Nyala Premium, Delight, Elleni and Gisilla), some legally imported international brands (Rothmans and Marlboro in 2018) and Winston—introduced by the NTE in 2019 as legal. These packs mostly complied with the Ethiopian labelling law. In 2019, the NTE stopped importing cigarettes. Therefore, all foreign brands (with the exception of Winston) were considered illicit in the 2022 survey. In our 2018 price data we only had seven Rothmans and two Marlboros, both considered legal in that year. None of these brands were detected in 2022, so their prices did not impact the prices of illegal brands in 2022.

### Data analysis

The data were analysed using Stata SE V.16.0. Descriptive statistics (frequencies, mean, median, SD) were presented to show the trend in cigarette price from 2018 to 2022 by brand, legal status, store type and location. We used the consumer price index to calculate the real value of cigarette prices.

To determine the difference between legal and illegal price changes before and after the tax increase, we employed a difference-in-difference model^26^ using the following formula:

$Price=B0+B1\;Legalbrand+B2\;Post-tax+B3\;Legalbrand*post-tax+B4\;store_{t}ype+B6\;town$

The dependent/outcome variable ‘Price’ is continuous and defined as the nominal price of a single stick cigarette in ETB as reported by the retailers.

The independent/control variables are defined as follows:

‘Legalbrand’ is a dummy variable that has a value of 1 if the cigarette is legal, 0 otherwise.

‘Post-tax’ is a dummy variable that has a value of 1 if the price observation was made in 2022, 0 otherwise.

‘store_type’ is a categorical variable.

‘town’ is a categorical variable.

The interaction term Legalbrand*post-tax describes the difference in prices between the legal and illicit brands after the tax increase.

## Result

Table 1 summarises the trend of cigarette prices between 2018 and 2022 based on an analysis of the 1774 observations. Of these, 1070 and 704 observations were recorded in the 2018 and 2022 survey years, respectively. Overall, 70.7% of the price observations belonged to legal cigarettes, and the remaining 29.3% were illicit brands. The most common brand is the domestically manufactured Nyala, but many illicit brands are also popular. In 2022, we noticed an increasing variety of illicit brands being available on the market: there were 10 illicit brands in 2018, but already 16 illicit brands in 2022. Among the legal products in 2018, legally imported Marlboro and Rothmans were the most expensive brands. In 2022, only NTE products were considered legal products, with Winston and Nyala Premium being the most expensive. Nyala and Nyala Premium were the only two legal brands found in both surveys. Green Apple and Benson & Hedges brands were the most expensive cigarettes in both the 2018 and 2022 surveys, even though other similarly priced brands appeared in 2022 (table 1).

**Table 1:** Frequency and average price (ETB) of single stick cigarettes by brand and survey year

Legal status	Brand	Year					Total observation	% change in the real price
		2018		2022				
		Observation	Nominal	Observation	Nominal	Real		
Licit	Delight	45	1.46	0	NA	NA	45	NA
	Elleni	0	NA	1	1.5	0.85	1	NA
	Gisilla	2	0.88	0	NA	NA	2	NA
	Marlboro	2	5	0	NA	NA	2	NA
	Nyala	799	1.29	318	2.63	1.49	1117	15
	Nyala Premium	52	1.45	15	2.7	1.53	67	5
	Rothmans	7	2.86	0	NA	NA	7	NA
	Winston	0	NA	13	2.73	1.54	13	NA
Illicit	Awsan	0	NA	4	2	1.13	4	NA
	Benson & Hedges	11	2.09	11	5	2.83	22	35
	Blues English	1	1	1	2.5	1.41	2	41
	Bon International	2	1	2	4	2.26	4	126
	Business Royals	58	0.78	49	3.08	1.74	107	123
	Ghamdan	17	0.75	20	1.92	1.08	37	45
	Gold Mount	2	1	15	3.23	1.83	17	83
	Gold Seal	0		82	2.12	1.2	82	NA
	Green Apple	4	3.25	12	4.67	2.64	16	−19
	MAC	0		1	8	4.52	1	NA
	Manchester	2	1.75	1	2.5	1.41	3	−19
	Oris	12	1.79	8	4.13	2.33	20	30
	Reds American	0		5	2.8	1.58	5	NA
	Rothmans Royals	54	1.25	2	3	1.7	56	36
	Shamlan	0		143	2.85	1.61	143	NA
	YNOT	0		1	2	1.13	1	NA
	*Total*	1070		704			1774	
Legal status (%)	Legal	84.8		49.3			70.7	
	Illicit	15.2		50.7			29.3	

ETBEthiopian birrNAnot available

The real prices of all legal brands increased from 2018 to 2022, with Nyala increasing by 15%. In nominal and real terms, a pack of Nyala increased by ETB27 and ETB4, respectively. The 2020 tax increase was ETB14 per pack.^8^ If we subtract the nominal tax increase from the nominal price, we get an ETB13 inflation-driven increase per pack of Nyala, or about a 50% increase. General inflation between 2018 and early 2022 was about 77%. This indicates that the NTE undershifted the tax increase to the price of the most popular brand Nyala. The undershifting is even larger on Nyala Premium.

The prices of illicit brands also increased in real terms except for Green Apple and Manchester, although Manchester brand has few observations. The most frequent illicit brand in both surveys was Business Royals whose price increased by 123% in real terms between the two surveys.

Table 2 shows the prices across different cities in 2018 and 2022. The real prices of licit cigarettes increased over time in all towns except Bahir Dar. The real prices of illicit cigarettes also increased in most places except for Assosa, Bahir Dar and Logiya. Nominal prices increased in all cities except for illicit brands in Bahir Dar.

**Table 2:** Average price of single stick cigarettes across towns and survey years

Town	Survey year								% change in real prices (licit)	% change in real prices (illicit)
	2018			2022						
	Observation	Licit	Illicit	Observation	Licit		Illicit			
		Nominal	Nominal		Nominal	Real	Nominal	Real		
Overall	1070	1.26	1.17	704	2.63	1.49	2.84	1.6	18	37
Addis Ababa	189	1.21	1.6	103	2.83	1.6	3.17	1.79	32	12
Assosa	162	1.1	3	51	2.39	1.35	3.96	2.24	23	−25
Bahir Dar	184	1.65	3.75	70	2.55	1.44	2.58	1.46	−13	−61
Dire Dawa	88	0.99	0.88	78	2	1.13	2.89	1.63	14	86
Gambella	93	1.03	NA	34	2.63	1.49	NA	0	44	NA
Ginir	0	NA	NA	39	2.45	1.38	2.55	1.44	NA	NA
Hawassa	58	1.14	NA	137	2.85	1.61	3.6	2.03	41	NA
Jigjiga	78	1	1.19	12	NA	NA	4.5	2.54	NA	114
Logiya	93	1.3	1.75	100	2.41	1.36	2.08	1.18	5	−33
Mekele	51	1.31	NA	0	NA	NA	NA	NA	NA	NA
Moyale	74	1.2	NA	0	NA	NA	NA	NA	NA	NA
Woldia	0	NA	NA	80	2.32	1.31	2.93	1.66	NA	NA

NA, not available

Table 3 presents the mean and the median cigarette prices by legal status and the type of outlet. Both legal and illicit products had higher prices in 2022, with illicit product prices growing faster than the legal ones. The prices of cigarettes sold in kiosks grew faster compared with those sold via street vendors. The real prices declined among street vendors, but this was driven by the change in the type of brands they were selling in 2018 and 2022.

**Table 3:** Average price of single stick cigarettes (ETB) by legal status and type of store

Variables	Summary statistics	Survey year			% real change
		2018	2022		
		Nominal	Nominal	Real	
Overall	Mean	1.25	2.74	1.55	24
	95% CI	(1.23 to 1.27)	(2.69 to 2.79)		
	Median	1.25	2.5	1.41	13
	SD	0.38	0.67	0.38	
	Observation	1070	704	397.78	
Licit	Mean	1.26	2.63	1.49	18
	95% CI	(1.24 to 1.28)	(2.60 to 2.67)		
	Median	1.25	2.5	1.41	13
	SD	0.34	0.36	0.2	
	Observation	907	347		
Illicit	Mean	1.17	2.84	1.6	37
	95% CI	(1.09 to 1.26)	(2.75 to 2.93)		
	Median	1.25	3	1.7	36
	SD	0.56	0.87	0.49	
	Observation	163	357		
Kiosk	Mean	1.21	2.7	1.53	26
	95% CI	(1.19 to 1.23)	(2.65 to 2.76)		
	Median	1.25	2.5	1.41	13
	SD	0.33	0.66	0.37	
	Observation	1009	509		
Street vendor	Mean	1.91	2.83	1.6	−16
	95% CI	(1.64 to 1.72)	(2.73 to 2.93)		
	Median	2	3	1.7	−15
	SD	0.49	0.7	0.4	
	Observation	61	195		

CIconfidence intervalETBEthiopian birrSDstandard deviation

### Tax impact on cigarette price

Both the bivariate and the multivariate analyses show that legal cigarettes were a bit more expensive before the tax increase compared with their illicit counterparts, while the opposite was true after the tax increase. This change is statistically significant at p<0.01 (table 4). The price of both legal and illegal cigarettes increased significantly in 2022.

**Table 4:** Results of the difference-in-difference model

Variables	Bivariate			Multivariate		
	Price	SE	P value	Price	SE	P value
Before tax increase						
Illicit (I)	1.173			0.714		
Licit (L)	1.262			0.775		
Difference (I−L)	0.088	0.045	0.05	0.06	0.119	0.61
After tax increase						
Illicit	2.84			2.371		
Licit	2.633			2.076		
Difference	−0.207	0.05	<0.01	−0.295	0.051	<0.01
Difference-in-difference	−0.296	0.067	<0.01	−0.355	0.124	<0.01

SEStandard Error

## Discussion

This study demonstrates the response of legal and illegal cigarette prices to a tax increase in Ethiopia. We used transaction price data obtained from retailers before and after the 2020 tax increase in geographically diverse parts of Ethiopia.

Even though prices differ across cities, the legal, locally manufactured cigarettes are often cheaper than the illegal foreign brands, except for legally imported Rothmans and Marlboro brands in 2018. Illegal flavoured cigarettes that are banned in Ethiopia (eg, Green Apple) were among the most expensive cigarettes in both 2018 and 2022. Cigarettes sold by street vendors were more expensive compared with those sold in kiosks, but this price gap narrowed over time.

Overall, the average price of legal cigarettes was higher in 2018 than that of illicit cigarettes, while the opposite was true in 2022. The high prices of legal cigarettes in the 2018 survey were somewhat influenced by Rothmans and Marlboro brands that used to be imported by the NTE. However, the NTE stopped cigarette imports in 2019 as it began to manufacture its own foreign brand Winston priced similarly to Rothmans.^20^

Illicit cigarettes have been found more expensive compared with their legal counterparts in other countries.^27 28^ This signals that price/tax is not the only motivator for buying illegal products.

After the change in tax structure and the tax increase in 2020, cigarette prices of both legal and illegal brands went up in both nominal and real terms, with prices of illegal brands growing faster. Thus, the tax increase influences the prices of legal products and illegal ones. This has been observed elsewhere.^29^ Illicit brand price increase may have been additionally motivated by the cigarette import ban.

Despite the 2020 tax increase, Ethiopia is far from the recommended 75% minimum tax share in price (the 2020 tax share in price was 54%^17^) and cigarettes continue to be cheap: a single cigarette stick can be purchased for an average price of ETB2.74 (US$0.05) or for ETB55 (US$1.00) a pack. This is much lower than the price of most cigarette brands sold in neighbouring Kenya, for example, where a pack costs about US$2.32.^30^

Given the high level of inflation, the specific component of tax needs to be frequently adjusted for inflation. Just between 2018 and 2021 inflation was approximately 77%, and the 2022 inflation rate is expected to be 33.64%.^31^ The specific tax of ETB8 put in place in 2020 is worth only ETB4.73 in 2022. The current law gave the Minister of Finance the power to increase the excise tax up to 10% at least once a year to account for inflation.^15^ Unfortunately, this has not happened, as the industry is spreading misinformation about the impact of the increased tax on illicit trade.^32^ Yet, the prices of illegal brands are higher compared with the legal ones. This is similar to the situation in Vietnam where a study found that the size of illicit cigarette market declined after a tax increase.^28^ Therefore, the 2020 tax increase cannot be a reason for the persistence presence of illicit cigarettes. Since smokers in Ethiopia are willing to pay a premium for foreign brands that are now all illegal (except for domestically produced Winston), and in view of the apparent inability to prevent the inflow of illicit foreign brands, the government should at least consider opening the market to legal imports in order to possibly capture some revenue it is now losing.

WHO predicted that the tax increase would result in a 54% cigarette price increase. We detected only a 24% real price increase between 2018 and 2022. Hence, the tax increase may not bring the intended outcomes in terms of smoking prevalence. The revenue goal is also undermined by the failure to adjust the specific tax rate for inflation.

Our study indicates that the NTE undershifted the tax increase. This may lead to lower profits unless the NTE found a way to lower its tax liability. Given the current ownership of the NTE (owners are from Japan and Yemen) and the fact that the second most popular brand in 2020 was illicit Shamlan brand from Yemen, we are leaning towards the second hypothesis.

### Strengths and limitations

We could not determine price changes by brands since many of them disappeared from our 2022 sample while new ones appeared. The appearance of new brands is a significant feature of the illicit cigarette market in Ethiopia. We also did not collect prices in rural areas due to budget and time constrains. Despite these limitations, our findings provide important evidence vis-à-vis the illicit cigarette market in Ethiopia.

## Conclusion

The price of both legal and illicit cigarettes increased after the 2020 tax increase. This is good news for public health.

We found that customers are willing to pay more for illicit imported cigarettes than the legal local products. Therefore, there is a space for the government to further raise excise taxes to achieve better health and increase domestic revenues. When raising tax rates, the government should consider the specific tax component for inflation to make sure that its real value does not diminish over time. Strengthening tax administration, including better supply chain controls, would be beneficial for both the public health and the tax revenue.

## Data Availability

Data are available upon reasonable request.
